# Health and Nutrition Studies Related to Cereal Biodiversity: A Participatory Multi-Actor Literature Review Approach

**DOI:** 10.3390/nu10091207

**Published:** 2018-09-01

**Authors:** Francesco Sofi, Monica Dinu, Giuditta Pagliai, Leonardo Cei, Giovanna Sacchi, Stefano Benedettelli, Gianluca Stefani, Edneia Gagliardi, Paola Tosi, Riccardo Bocci, Bettina Bussi, Giuseppe de Santis, Ismael Rodriguez y Hurtado, Patrick de Kochko, Pierre Riviere, María Carrascosa-García, Ignacio Martínez

**Affiliations:** 1 Department of Experimental and Clinical Medicine, School of Human Health Sciences, University of Florence, 50121 Florence, Italy; francesco.sofi@unifi.it (F.S.); mdinu@unifi.it (M.D.); giuditta.pagliai@gmail.com (G.P.); 2 Don Carlo Gnocchi Foundation, 20121 Milan, Italy; 3 Department of Land, Environment, Agriculture and Forestry, University of Padua, 35020 Padua, Italy; leonardo.cei@phd.unipd.it; 4 Department of Economics and Management, University of Florence, 50121 Florence, Italy; gianluca.stefani@unifi.it; 5 Department of Agrifood Production and Environmental Sciences, University of Florence, 50121 Florence, Italy; stefano.benedettelli@unifi.it; 6 Agri-Food Economics and Social Science, School of Agriculture Policy and Development, University of Reading, Reading RG66AH, UK; e.gagliardi@reading.ac.uk; 7 Crop Production, School of Agriculture Policy and Development, University of Reading, Reading RG66AH, UK; p.tosi@reading.ac.uk; 8 Rete Semi Rurali, 50018 Florence, Italy; r.bocci@casignano.it (R.B.); bettinabussi@semirurali.net (B.B.); giuseppe.desantis@semirurali.net (G.d.S.); 9 Réseau Semences Paysannes, 47190 Aiguillon, France; ismael@semencespaysannes.org (I.R.y.H.); patrick@semencespaysannes.org (P.d.K.); pierre@semencespaysannes.org (P.R.); 10 Red Andaluza De Semillas Cultivando Biodiversidad, 41015 Seville, Spain; maria_carrascosa@riseup.net (M.C.-G.); imarnog@gmail.com (I.M.)

**Keywords:** cereal biodiversity, ancient grains, whole grains, metabolic syndrome, gluten-related disorders, inflammatory parameters, participatory multi-actor approach

## Abstract

Recently, a large and growing body of literature has investigated the health potential of different wheat species. In particular, a considerable number of studies dealing with nutritional aspects has grown up around the theme of the recovery of ancient wheat varieties (species that have remained unchanged over the last hundred years). According to several studies, indeed, ancient varieties present a healthier nutritional profile than modern ones. In the framework of the European project “CERERE, CEreal REnaissance in Rural Europe: embedding diversity in organic and low-input food systems”, this paper aimed to review recent research on the issue of health and nutritional cereal systems by adopting an innovative and participatory multi-actor approach which involved practitioners along with researchers. The participatory approach is the main innovation and peculiarity of this literature review. Nevertheless, the review highlights the many positive effects derived from eating whole and ancient grains such as a significant reduction in the risk of chronic diseases such as cancer, cardiovascular disease, and also a more favorable long-term weight management and increase in satiety. This review may be considered as a fruitful starting point that integrates research results to foster current and future healthier and sustainable practices in cereal systems.

## 1. Introduction

Consumed by billions of people, cereal grains are the main staple food in many diets, providing a large percentage of daily energy intake. In the context of a balanced diet, cereals—especially when consumed as a whole—represent a healthy source of multiple nutrients, dietary fibers [1], and bioactive peptides with anticancer, antioxidant, and antithrombotic effects [2]. It has been suggested that whole wheat flour can also modulate the metabolic activity of the gut microbiota to increase the production of beneficial metabolites [3,4,5]. Based on the increase in worldwide mortality attributable to diet-related chronic diseases, in recent years, there has been a growing interest in identifying cereals with a greater health potential. In particular, ancient grains (defined as those grains that have remained unchanged over the last hundred years) have gained interest since several studies have suggested that they are higher or characteristic in some components such as minerals and polyphenols [6,7,8]. In light of this, efforts are being made to induce people to replace refined cereals with whole and ancient grains [9,10]. In addition, researchers are trying to improve the nutritional proprieties of the most widely used cereal products such as bread or tortillas through the incorporation of legumes and rye flour, flaxseeds, and other ingredients [11,12,13,14]. Likewise, sourdough has been successfully applied to improve the quality of gluten-free bread [15].

The purpose of this paper was to review recent research on the health and nutritional aspects of whole and ancient grains following an innovative participatory approach that involved academics as well as practitioners according to the European Union “multi-actor approach” guidelines. This means that partners with complementary types of knowledge—scientific, practical, and other—join forces in the project activities from beginning to end.

This research was indeed carried out as part of the European Horizon 2020 thematic network “CERERE, CEreal REnaissance in Rural Europe: embedding diversity in organic and low-input food systems. CERERE” is an on-going three-year project financed by the European Commission. It involves nine European Countries characterized by very different features in their agricultural and food sectors and history. The project focuses on the cereal sector due to its importance in the European agriculture and tradition, and deals with several stages of the supply chain pursuing different objectives. In particular, as for agricultural production practices, it aims to improve and manage the agro-biodiversity of European cereal systems as well as promote the adoption of low-input production practices. At the processing level, it encourages the rediscovery of traditional techniques necessary to work with non-conventional raw materials (e.g., ancient varieties) as current industrial methods are not able to cope with them. Some of its wider objectives are the promotion of healthy food systems and the creation and reinforcement of networks comprising all of the actors directly or indirectly involved in the production process and in its improvement in terms of ecological, economic, and social sustainability. Indeed, such networks are able to foster cooperation and the transmission of both scientific and practical knowledge, favoring the adoption of good practices throughout different production stages as well as through different supply chains, actually promoting innovation.

According to the project’s objectives, the composition of the project actors is highly heterogeneous. Among the 13 partners, there are providers of scientific knowledge such as universities and research centers, extension and advisory centers, farmers’ networks, and training and communication organisms. This heterogeneity allows for the exploitation of different kinds of knowledge and experiences and opens up participatory debates where innovation can emerge. Indeed, since food system innovation is embedded in social, cultural, and economic contexts and in changing societal demands for quality and healthy food, participatory and multi-actor approaches, which bring together researchers, food system practitioners (from farmers to food manufacturers), and consumers, are crucial to ensuring that the innovations proposed towards diversification and sustainability are appropriate for each context and incorporate values such as local identities, nutritional quality, and health.

In this sense, the novelty of this research lies specifically in the participatory approach itself. The scope of the paper, indeed, was to assess the state of the art of current research on several topics to shed light on both the practical and scientifically relevant issues. As a result of this approach, the paper was not designed as a review or a systematic review that normally identifies and synthesizes the body of the relevant literature of specific topics. On the one hand, this is a strength, as the selected literature was due to construct relevant issues to the stakeholders. On the other hand, the coverage of themes and papers mirrored the specific interests, value systems and points of view of *CERERE* consortium participants.

As a consequence, the results of the participatory review are not to be considered as a final goal, but they will be coupled, at a later stage of the project, with real supply chain case studies to gain useful insights into the development and the functioning of such processes.

Due to the large scope of the project, three different focus areas were identified and a specific literature review was performed for each. This paper covers the “Health and Nutrition focus area, while two other papers are dedicated to Rural Development and Agronomy and Food Processing”.

The paper is organized as follows: Section 2 covers the methodological aspects of the study. Section 3 is concerned with the discussion of the results, while Section 4 presents our conclusions.

## 2. Methods

The methodology used in the literature review can be conceptually divided into three steps: study retrieval, study screening, and content analysis.

### 2.1. Study Retrieval

To retrieve the studies suitable for inclusion in the review, we searched two bibliographic databases: PubMed and Scopus, performing the search in title, abstract, and keywords. The keyword selection was performed in a participatory way during a project meeting where all partners were involved. Each partner suggested several terms that were later refined and/or aggregated to limit their number and to produce consistent and effective search keywords. We performed the search by clustering the actual keywords in groups of concepts and structuring search strings, combining the newly created clusters through Boolean operators. The following example illustrates the structure of a standard search string, where keywords included in brackets belong to the same group of concepts.

(wheat OR rye OR oat OR spelt OR barley OR bread OR pasta) AND (“whole wheat” OR ancient OR landraces OR “traditional varieties” OR “heritage varieties”) AND (health OR nutrition OR diet) AND (diabetes OR “glycemic index”)

This search method provided almost all of the studies included in the review. However, partners were encouraged to suggest other material to add, based on quotes they found in reviewing the initial set of papers or their previous knowledge.

### 2.2. Study Screening

Due to the high number of retrieved references, the very first selection was based on titles and was intended to immediately exclude works clearly irrelevant to the project objectives. Moreover, we rejected entries that did not fulfil some of the basic criteria such as the year of publication, the geographical location, and the language (Table A1 in Appendix).

A second step consisted of further selecting studies investigating the abstracts’ content and its accordance with the project covered topics. During this phase, the nature (qualitative/quantitative) of each study was also assessed.

Finally, the selected references were evaluated with respect to methodological and relevance issues through a full-paper analysis. Methodological criteria differed for qualitative and quantitative studies and are reported in Table A2 and Table A3, while the relevance criteria are illustrated in Table A4. The compliance with both kinds of criteria was assessed through YES/NO questions and the evaluation was on a 1–5 Likert scale of several statements.

**Figure 1:**
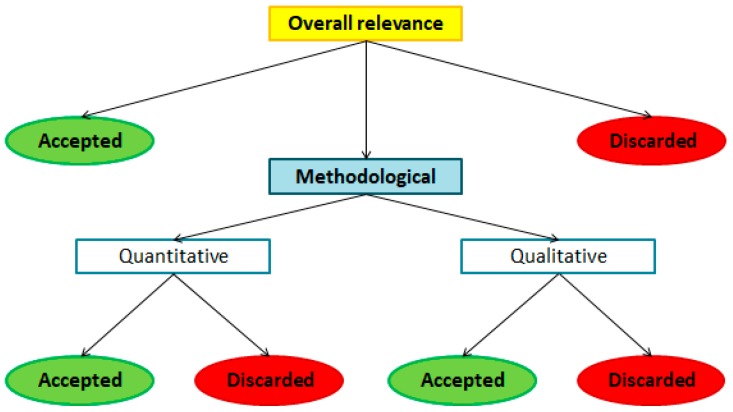
Graphic of the acceptance process of the papers.

At the end of the evaluation, each study received an overall relevance score which, combined with the methodological assessment results, determined its final acceptance/withdrawal. Rules followed in the acceptance process are schematically shown in Figure 1.

First, the score assigned by the reviewers to the “overall relevance” section was considered. As the score was on a 1–5 scale, we set the neutral value, i.e., 3, as the acceptance threshold. Therefore, a paper was included in the review when receiving a mean overall relevance score higher than 3 (the paper is considered quite relevant or totally relevant for the project), and discarded if its score was 1 or 2 (the paper is considered quite irrelevant or totally irrelevant). For those papers where the relevance evaluation did not provide clear results (mean score equal to 3), we decided to determine the inclusion using methodological aspects by combining the results of the methodological form sections. Specifically, a quantitative study was accepted if it received a mean score for the “statements” section (see Table A3) higher or equal to 3, and it received for each statement a score equal to 2 or higher. Moreover, at least three out of the four “questions” in the quantitative form needed to receive a “YES” answer. Acceptance of the qualitative studies followed the same rules, with the exception of the “questions” part, since questions were not included in the qualitative form (see Table A2).

### 2.3. Content Analysis

**Figure 2:**
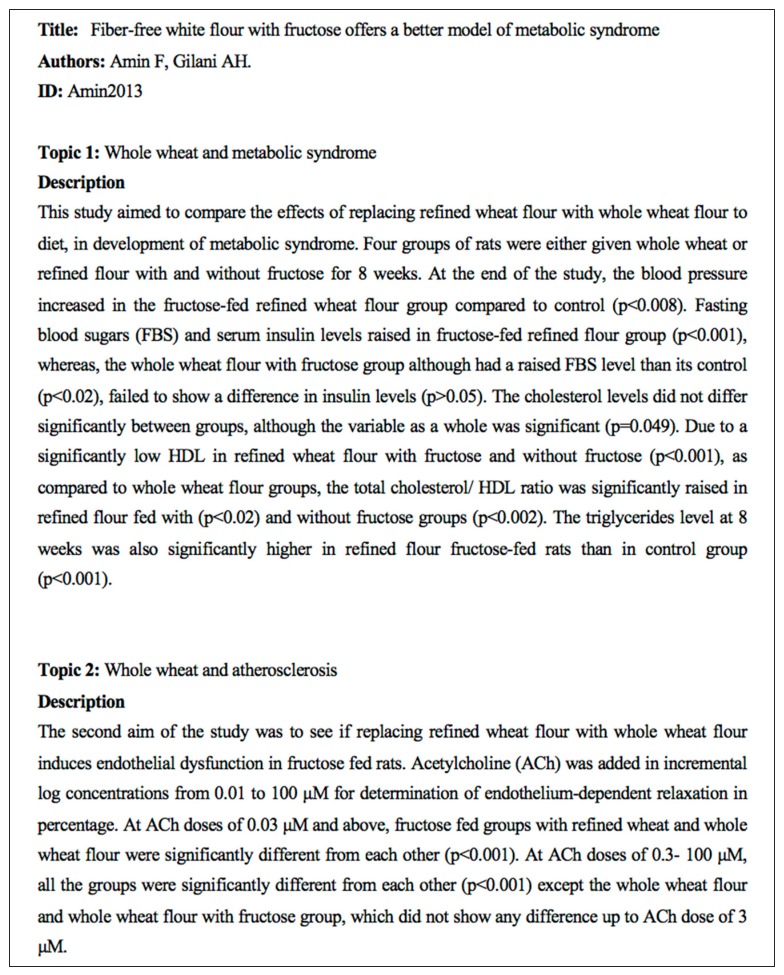
Example of the final structure of the text form arisen from the content analysis.

After the final list of papers was compiled from the combined implementation of the methodological and relevance selections, a content analysis was performed. We provided each academic partner with a share of papers, taking care to assign some papers to more than one reviewer to check for accordance in the analysis. The reviewer had to produce a form (like the one in Figure 2) for each study that highlighted the main topics covered and provided a synthetic description of them.

To facilitate the discussion, similar topics were aggregated in clusters representing different research areas. This was done in a participative process involving all of the project partners (both academic and non-academic).

## 3. Discussion of Results

### 3.1. Quantitative Aspects of the Literature Review

The initial search in bibliographic databases produced 609 references, which was reduced to 272 after applying the first filtering based on title, year, and language. The selection based on the abstracts’ contents provided 100 records on which to apply the full-paper screening. The entire process ultimately ended with the content analysis of 48 papers, two of which were added according to the partners’ suggestions.

**Figure 3:**
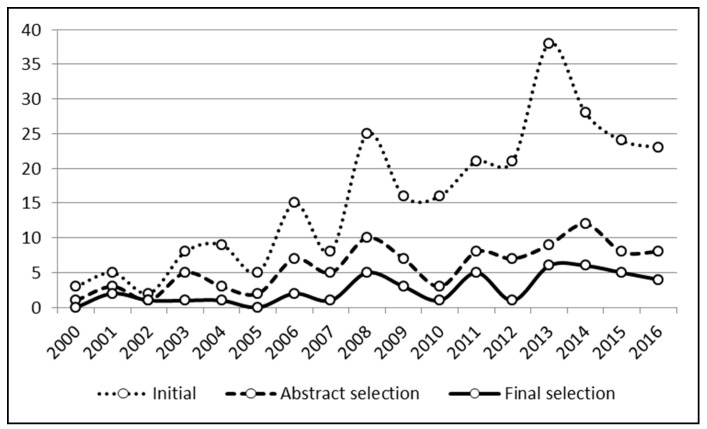
Number of published papers per year at different review process stages.

Figure 3 explores the change over time in the number of papers initially retrieved from the databases and after the different screening phases. Published papers on the topic have clearly increased in recent years. However, the process produced a higher discard rate for recent papers, as suggested by the flatter lines representing the paper passing through the filtering steps.

As anticipated in the methodological section, quantitative and qualitative papers were evaluated using different methodological criteria. The former type was the most represented in the set of papers successfully passed through the abstract selection (74%) and approximately the same ratio was preserved at the end of the full process. With respect to the quality evaluation, the quantitative studies performed better, receiving an average score in the “statements” section of the evaluation form of 3.54, compared to the 3.23 received by their qualitative counterpart. Despite this score being considered quite high in the 1–5 scale used for the evaluation, the “questions” provided quite contrasting results, displaying only 51.5% of YES answered questions.

**Figure 4:**
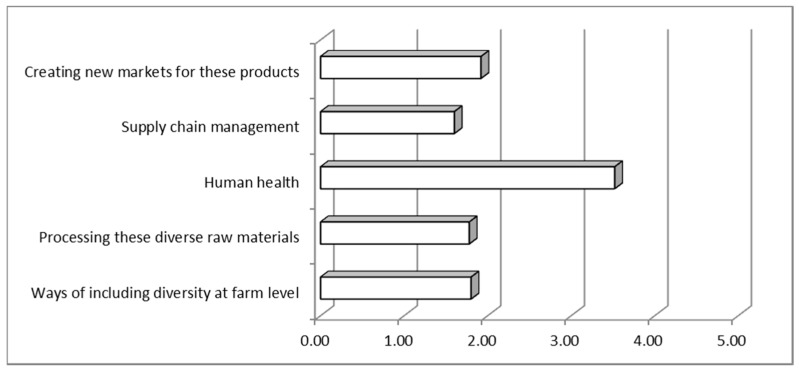
Scores assigned to the evaluated papers in the “practical implication” screening form.

The relevance screening highlighted that the studies’ “practical implications” concern, as expected, was mostly in the human health sphere, but other topics also gained a not irrelevant coverage (Figure 4). However, these results should be taken with caution since, according to the evaluation, only 20% of the papers were deemed to address practical problems.

Another interesting point worth noting is the accordance between the methodological and the relevance quality. To do this, a contingent table was provided (Table 1) where the quality classes were derived from those used in the evaluation process (see Figure 1 in Section 2.2). The classes used in Table 1 refer to the categorization made to determine the acceptance/rejection of the screened papers (see Figure 1). Specifically, for the relevance evaluation, Class 1 included papers with an “overall relevance” score greater than 3, Class 2 were those with a score equal to 3, and Class 3 were those with a score smaller than 3. On the other side, methodological Class 1 coincided with the paths leading (in Figure 1) from the methodological screening to the final acceptance, the other studies being included in Class 2.

Inspection of the table reveals a general discordance between the two types of evaluation since the majority of methodological “Class 1” papers was assigned to the relevance “Class 3”, while conversely, records included in the second methodological class were quite evenly split between the first and the third relevance classes.

### 3.2. Discussion of Literature Review Contents

Eaten in the recommended amounts, whole grains have been associated with a significant reduction in the risk of chronic diseases such as cancer [16], metabolic syndrome [17] and hypertension [18,19], more favorable long-term weight management [20], and an increase in satiety [21]. Each of these points will be considered herein.

#### 3.2.1. Glycemic Profile

The effects of whole and ancient grains on the glycemic profile have been investigated in both animal and human studies. A study that investigated the effects of eating four commonly consumed whole grains in diabetic control and progression in rats reported modest benefits [22]. The replacement of whole wheat with refined wheat flour, on the other hand, has caused hyperinsulinemia and hyperglycemia [23]. Ancient wheat diets have caused a downregulation of key regulatory genes involved in glucose and fat metabolism and a consequent reduction in insulin levels in a study involving Zucker diabetic fatty rats [24]. Likewise, glycaemia was significantly lower in rats fed with ancient Kamut^®^ khorasan when compared to those fed a standard diet [25]. Consistent with these results, a replacement diet with products made with Kamut^®^ khorasan wheat reduced fasting glucose and insulin levels in healthy participants [6] as well as in diabetic patients [26] and participants at high-risk for cardiovascular disease [27]. Even the ancient varieties “Verna”, “Gentil Rosso”, and “Autonomia B” have led to a significant reduction in glycemia [28]. For whole grains, the results are conflicting. While some studies have not found significant differences between white and whole bread on the glycemic profile [19,29], others have suggested that the consumption of whole wheat products leads to lower levels of fasting glucose [28,30,31] and insulin [32].

#### 3.2.2. Lipid Profile

Several animal studies have reported a positive effect on whole grains on cholesterol and triglyceride levels [4,23,33]. Ali [34] investigated the effects of kishk, a mixture of dried fermented wheat/milk, also finding a significant improvement in cholesterol, LDL cholesterol, and triglyceride levels. In human studies, a significant reduction in cholesterol and triglycerides was observed for whole wheat [30] and barley [35]. The positive effects were also observed for ancient grains. The consumption of products made with Kamut^®^ khorasan and the ancient varieties “Verna”, “Gentil Rosso”, and “Autonomia B” has led to a significant improvement in total and LDL-cholesterol [6,26,27,28]. In contrast, the consumption of white bread has been correlated with an increase in triglyceride levels [19].

#### 3.2.3. Oxidative Stress and Inflammatory Parameters

The consumption of whole wheat bread containing bioprocessed bran with a greater bioavailability of ferulic, vanillic, sinapic, and 3,4-dimethoxybenzoic acids showed anti-inflammatory properties in an ex vivo LPS-challenge [36]. Positive effects on various oxidative and inflammatory parameters have also been reported for ancient grains in both animal and human studies. The rats fed Kamut^®^ pasta showed a lower oxidative state under basal conditions and a better response to exogenous oxidative stress, partly responsible for the increased activity of liver antioxidant enzymes [37]. In another study, Kamut^®^ khorasan bread-fed rats had a better response to stress than those fed wheat durum, especially when a sourdough bread was provided [25]. Positive effects on oxidative stress and inflammatory parameters were also observed in a study with rats fed Kamut^®^ khorasan biscuits [38]. Regarding human studies, clinical studies have suggested that the consumption of products with Kamut^®^ khorasan improved the oxidative status and levels of various inflammatory cytokines [6,26,27,39].

#### 3.2.4. Gluten-Related Disorders

Gluten and other wheat proteins are involved in the development of conditions such as celiac disease, non-celiac gluten sensitivity, and intestinal bowel disease. It has been shown that the consumption of ancient grains is not safe for patients with celiac disease [20,40], but may provide some benefits to individuals with irritable bowel syndrome. For example, a randomized clinical trial found a significant reduction in the severity of irritable bowel syndrome symptoms after consumption of Kamut^®^ khorasan products [39]. This result is promising because gluten-related disorders are showing a rapidly changing trend, especially in the Western world. Some authors have suggested that Western people are more inclined than Africans and Asians to face the side effects related to the consumption of modern wheat varieties [41].

## 4. Conclusions

The present paper aimed to review the research on the effects of alternative cereal systems by adopting an innovative and participatory multi-actor approach which involved both practitioners and researchers. The participatory approach, along with the consequent methodological parameters applied, was the main innovation and peculiarity of this literature review. On the other hand, the main limitation of the study was represented by the coverage of papers, which was not complete as only those deemed relevant to the project partners were considered. Nevertheless, the systematic approach embraced and the adoption of specific selection criteria assured the overall transparency of the whole process. In a sense, this review may be considered as a starting point in integrating research results to foster current and future healthier and sustainable practices in cereal systems.

Generally speaking, the results of the papers covered by this review suggest that whole and ancient grains are increasingly recognized for the nutrients they provide and the complex role they play in promoting health [8]. The macro- and micronutrients along with the phytonutrients present in their seeds seem to synergistically contribute to reducing the risk of several chronic diseases such as cardiovascular disease, diabetes, obesity, and certain cancers. While not all intervention studies have shown beneficial effects for the consumption of whole or ancient grains, none of these studies have shown negative impacts on the health outcomes tested. Most findings derived from animal and human studies actually suggested that whole and ancient grains ameliorate glycemic and lipid status as well as pro-inflammatory and anti-oxidant parameters [8]. Evidence is also emerging for the role of ancient grains in reducing the severity of gastro-intestinal and extra-intestinal symptoms in patients with irritable bowel syndrome.

In conclusion, the results of the dietary intervention trials available in the literature allows us to suggest the possible beneficial effects on human health of ancient grains. Therefore, as a large proportion of the population could benefit from eating more whole and ancient grains, major efforts should be made to encourage further and larger studies.

## Figures and Tables

**Table 1:** Contingent table (methodological quality vs relevance quality).

		Relevance				
		Class 1	Class 2	Class 3	Total	
Methodological	Class 1	2	8	11	21	Qualitative
	Class 2	2	0	3	5	
	Total	4	8	14	26	
	Class 1	16	13	29	58	Quantitative
	Class 2	5	3	8	16	
	Total	21	16	37	74	
	Class 1	18	21	40	79	Overall
	Class 2	7	3	11	21	
	Total	25	24	51	100	

## Appendix A

**Table A1:** Eligibility Screening.

Study ID			Paper_1	Paper_2	Paper_3	Paper_n
Questions	Is the study written in English, French or Spanish?	YES				
		NO				
	Are the areas interested by the study located in developed countries?	YES				
		NO				
	Has been the study published after 2000?	YES				
		NO				
Type of the study	Published article					
	Abstract/Presentation					
	Book/Book chapter					
	Technical/progress report					
	Working paper					
	Unpublished dissertation					
	Other (specify)					
Focus area of interest of the study	Agronomy and Food Supply Chain					
	Nutrition and Health					
	Rural Development					
Quantitative or qualitative study	Quantitative					
	Qualitative					

**Table A2:** Methodological Screening: Qualitative.

Study ID		Paper_1	Paper_2	Paper_3	Paper_n
Data collection method	Questionnaire				
	Secondary analysis				
	Interviews and/or focus groups				
	Literature review				
	Other (specify)				
Research strategy	Survey				
	Single case study				
	Multiple case study				
	Theoretical				
	Literature study				
	Other (specify)				
Participatory approach	YES				
	NO				
Statements(Assign to each a score from 1—strongly disagree—to 5—strongly agree)	The study’s objectives are clearly stated				
	The sample size is large enough and enough variety is present in respect to the most important variables (gender, farmers, retailers, consumers)				
	The data collection method is clearly defined				
	The method used in analyzing data is thoroughly explained				
Additional notes (and any additional comment that you deem necessary to assess the study, for example about the soundness of the theoretical references of the study)					

**Table A3:** Methodological Screening: Quantitative.

Study ID			Paper_1	Paper_2	Paper_3	Paper_n
Type of the study		Experimental study				
		Observational study				
		Review				
Questions	Are the study objectives and research questions clearly stated?	YES				
		NO				
	Are hypothesis thoroughly defined?	YES				
		NO				
	Which is the experimental design of the study (if applicable)?					
	Is the sample large enough according to the study objectives?	YES				
		NO				
	Has the sample the proper composition (gender, age…) according to the study objectives?	YES				
		NO				
Statements (Assign to each a score from 1—strongly disagree—to 5—strongly agree)	The data collection method is exhaustively explained					
	The data collection method is reliable (no measurement errors)					
	The method offers valid measures (they assess what it purports to measure)					
	The variables are clearly defined					
	The analytic/statistical method used is consistent with the study objectives					
	Results answer to all study questions					
	Study’s conclusion comes directly from the data collected by the study					
Additional notes (and any additional comment that you deem necessary to assess the study, for example about the soundness of the theoretical references of the study)						

**Table A4:** Relevance Screening.

Study ID			Paper_1	Paper_2	Paper_3	Paper_n
Scope of the study	Traditional food staff					
	New healthy products					
	Farming					
	Processing					
	Consumption					
	Other (specify)					
Questions	Is the study addressing practical problems?	YES				
		NO				
	Is a participatory approach in place?	YES				
		NO				
Practical implications	Ways of including diversity at farm level					
	Processing these diverse raw materials					
	Human health					
	Supply chain management					
	Creating new markets for these products					
	Other (specify)					
Overall relevance of the study	Overall relevance					
	Why?					
Case studies relation	Case studies to be coupled with the study					
	Why?					
